# Impact of sample size of birds processed per pen in research settings for performance and quality data

**DOI:** 10.1016/j.psj.2026.106879

**Published:** 2026-03-28

**Authors:** C.J. Maynard, K. Thompson, A. Mauromoustakos, C.M. Owens

**Affiliations:** a Center of Excellence in Poultry Science, University of Arkansas, Fayetteville, Arkansas, 72701, USA; b Agricultural Statistics Laboratory, University of Arkansas, Fayetteville, AR, 72701, USA

**Keywords:** Sample size, Broiler, Myopathy, Performance, Data simulation

## Abstract

Sampling procedures in scientific experiments have long been under scrutiny. The use of power testing and estimating replications has long been utilized as methods for designing experiments. In the same sense, simulation of reanalyzed data has become an approach for understanding on a more applied basis. In the current analysis, two recent trials were simulated 1000 times using a Monte Carlo design to represent performance and quality data of broilers reared to 52 and 56 days of age. Data consisted of male and female Ross 708 final live weights and then subsequently deboned into breast, tender, wing, and leg quarters to calculate yield. Additionally, broiler breasts were scored for woody breast and white striping. Pens contained 18, 21, 24, or 27 birds and all birds were processed at the time of sampling. Data for the current analysis were then analyzed as the maximum difference, as a percent, amongst all simulated pens and the true mean for all pens. Sample size was considered for each pen on the basis of increasing birds sampled per pen starting with 5 birds and concluding with 16 or 20 birds sampled per pen. Upon analysis, differences were observed between the performance characteristics and myopathy characteristics. Amongst live weight and carcass part yields, variance between the sample size of broilers processed was relatively minimized. Selecting any performance parameter expressed a maximum likelihood of 6.44% difference from the true mean when selecting 5 broilers for processing. However, myopathy differences did not follow a similar pattern. When assessing either woody breast or white striping, differences between sampled birds in the selected range could persist as high as 45% and 28%, respectively. This idea proves that myopathy characteristics are much harder to depict when sampling a smaller proportion of a pen rather than the culmination of a more robust sample size. In addition, a sample size of 5 to 20 birds consistently produces a tenfold difference when comparing performance and myopathy measures. Therefore, setting an acceptable threshold of possible error should be considered when sampling.

## Introduction

Recent investigation into proper sampling for meat quality in broilers has become prevalent. For decades, sampling discrepancies have been present between live performance studies (i.e., nutritional, management, physiology, immunology, etc.) and meat quality analyses (specifically myopathies). Generally, these studies focus on a sample of the population taking a range anywhere between 4-12 birds per pen to average performance gains (Coon et al., 1981; Kidd et al., 2004; Maynard et al., 2022c; Mullenix et al., 2022; Skinner et al., 1992). This number is typically based on processing availability, number of inherent replicates in the study, and the appropriate samples for building a statistical model. On the other hand, meat quality studies generally focus on harvesting an entire pen, or population, of birds to establish more accurate readings on a bird basis. Sampling of this nature is generally regarded as infeasible for large studies due to physical limitations of time and available resources in a scientific sampling. Although, analyses for breast meat quality are conducted on individual bird breast butterflies to allow for accurate analysis of the sample itself. Further complicating the disconnect between performance and myopathies, nutritional studies are now implementing myopathy analysis on their sample of birds to provide information that industry professionals are interested in quantifying. For this reason, the scope of this analysis was to define the appropriate number of birds that should be sampled (i.e., 4, 6, 8, etc.) from a pen to give an accurate and repeatable representation of performance and myopathy data in broilers.

## Materials and methods

Data analysis in this study used pre-existing data collected in trials where all animal experimentation was approved by the Institutional Animal Care and Use Committee at the University of Arkansas (protocols # 20016 and # 22056).

This analysis was warranted to better understand the relationship between performance and myopathy parameters. Therefore, a robust sampling set was first required to begin this analysis. However, attempting to reduce effects of treatment, data were selected from two studies that limited input variables. Trial 1 (females) provided inputs of sex, strain, and stocking density as the only effects presented in the analysis. Currently, female Ross 708 broilers were subjected to one of three commercial stocking densities with six replicates (total of 18 pens), grown to 52 days of age, and then processed to determine effects on live weight, carcass and part yields, and muscle myopathy scores. Pens consisted of either 21, 24, or 27 broilers per pen based on the study designed in a pre-existing experiment (Maynard and Owens, 2022). Trial 2 (males) provided inputs of sex, strain, and dietary supplementation of guanidinoacetic acid (GAA) as effects presented in the analysis. Male Ross 708 broilers were subjected to one of three diets with ten replicates (30 pens) consisting of a control, 0.06% addition of GAA to the control diet, and 0.12% addition of GAA to the control diet. Birds were grown to 56 days of age and then processed to determine effects on live weight, carcass and part yields, and muscle myopathy scores. Pens were originally placed with 25 broilers per pen and then culled to 18 broilers per pen to meet spatial requirements according to the study design in a pre-existing experiment (Maynard et al., 2023d).

Data from both studies included live weight, breast yield, wing yield, tender yield, leg quarter yield, and myopathy scores. Scores included white striping (**WS**) and woody breast (**WB**) on a 0-3 scale as described by Kuttappan et al. (2012b) and Tijare et al. (2016), respectively. Scores were then averaged to produce an overall mean score. Other meat quality attributes were not included for this analysis. Computing simulated pens first began with the establishment of the raw data from the original experiments. Raw data were sorted to establish a benchmark for sample size consideration (5-20), and then simulated to provide 1000 possible combinations of each individual bird of the original 48 pens and then recoded as a “new pen” utilizing a Monte Carlo design. Following simulation, these “new pens” were used to calculate a pen mean for each specified response (e.g., live weight, breast, tender, wing, and leg meat yield, and myopathy scores). Once individual means were obtained, “new pens” were sorted based on sample size, and tabulated to produce the 5% and 95% percentiles of the designated grand mean per pen. These two percentile estimates were then used to calculate the percent difference from the true pen mean (the actual observed mean). Once both percent differences were obtained, the maximum percent difference, whether that be from the 5% or 95% percentile, were collected and reported in the tables below (Table 1, Table 2). In addition, this maximum percent difference was then used to calculate a minimum and maximum range of potential values that could be obtained from the true pen means as provided in both experiments: [(Trial 1: live weight = 3.523 kg; breast yield = 25.23%; tender yield = 5.15%; wing yield = 7.57%; leg quarter yield = 22.07%; woody breast mean score = 0.74; white striping mean score = 0.95)(Trial 2: live weight = 4.716 kg; breast yield = 24.69%; tender yield = 4.81%; wing yield = 7.51%; leg quarter yield = 23.52%; woody breast mean score = 1.20; white striping mean score = 1.29)]. For sampling, 5 birds per pen was chosen as the minimum number sampled as it correlates to ∼20% of the pen as a baseline for typical processing in performance-based studies.

**Table 1:** Maximum percent difference in female Ross 708 performance and myopathy characteristics for different sample sizes based on a simulated 1000 pens of the true mean of all birds sampled per pen.

Sample Size^1^ (n=1000)	Live Weight Difference^2^	Live Weight Range^3^	Breast Yield Difference	Breast Yield Range	Tender Yield Difference	Tender Yield Range	Wing Yield Difference	Wing Yield Range	Leg Quarter Yield Difference	Leg Quarter Yield Range	Woody Breast Difference	Woody Breast Range	White Striping Difference	White Striping Range
5 birds	4.40	3.368-3.678	4.65	24.05-26.40	5.30	4.88-5.43	3.49	7.31-7.84	3.00	21.41-22.74	45.26	0.41-1.7	28.21	0.68-1.21
6 birds	3.83	3.388-3.658	4.06	24.20-26.25	4.65	4.91-5.39	3.08	7.34-7.81	2.70	21.48-22.67	40.28	0.44-1.04	24.53	0.71-1.18
7 birds	3.42	3.403-3.643	3.62	24.31-26.14	4.17	4.94-5.37	2.74	7.37-7.78	2.39	21.55-22.60	35.91	0.47-1.01	22.08	0.74-1.16
8 birds	3.10	3.414-3.632	3.27	24.40-26.05	3.83	4.96-5.35	2.52	7.38-7.76	2.21	21.59-22.56	32.65	0.50-0.98	19.82	0.76-1.13
9 birds	2.84	3.423-3.623	3.03	24.46-25.99	3.44	4.98-5.33	2.26	7.40-7.74	2.00	21.63-22.51	30.05	0.52-0.96	17.26	0.78-1.11
10 birds	2.57	3.432-3.614	2.76	24.53-25.92	3.14	4.99-5.32	2.07	7.42-7.73	1.81	21.67-22.47	26.90	0.54-0.94	15.60	0.80-1.09
11 birds	2.39	3.439-3.607	2.53	24.59-25.86	2.85	5.01-5.30	1.92	7.43-7.72	1.66	21.71-22.44	25.42	0.55-0.93	14.49	0.81-1.08
12 birds	2.18	3.446-3.600	2.33	24.64-25.81	2.62	5.02-5.29	1.74	7.44-7.71	1.53	21.74-22.41	22.43	0.57-0.91	13.45	0.82-1.07
13 birds	2.00	3.453-3.593	2.10	24.70-25.76	2.39	5.03-5.28	1.60	7.45-7.69	1.39	21.77-22.38	20.60	0.59-0.89	12.59	0.83-1.07
14 birds	1.81	3.459-3.587	1.92	24.74-25.71	2.17	5.04-5.27	1.44	7.46-7.68	1.29	21.79-22.36	19.35	0.60-0.88	11.72	0.84-1.06
15 birds	1.66	3.465-3.581	1.78	24.78-25.68	2.01	5.05-5.26	1.33	7.47-7.67	1.19	21.81-22.34	17.45	0.61-0.87	10.37	0.85-1.04
16 birds	1.52	3.469-3.577	1.61	24.82-25.63	1.86	5.06-5.25	1.20	7.48-7.66	1.05	21.84-22.31	15.46	0.63-0.85	9.12	0.86-1.03
17 birds	1.38	3.474-3.572	1.44	24.86-25.59	1.66	5.07-5.24	1.09	7.49-7.66	0.97	21.86-22.29	14.36	0.63-0.85	8.56	0.87-1.03
18 birds	1.22	3.480-3.566	1.30	24.90-25.55	1.49	5.08-5.23	0.97	7.50-7.65	0.84	21.89-22.26	12.55	0.65-0.83	7.80	0.87-1.02
19 birds	1.07	3.485-3.561	1.14	24.94-25.51	1.31	5.09-5.22	0.86	7.51-7.64	0.73	21.91-22.23	10.93	0.66-0.82	6.64	0.88-1.01
20 birds	0.85	3.493-3.553	0.88	25.00-25.45	1.06	5.10-5.21	0.69	7.52-7.63	0.61	21.94-22.21	8.68	0.68-0.80	5.69	0.89-1.00

1 Sampling of pens ranged from 5 to 20 broilers per pen processed to provide the variation possible between the subset being sampled and the true population mean.

2 Values in the difference category are presented as a percentage (%). Values are based on the maximum difference between the true observed mean of all birds processed per pen and the 5% or 95% percentiles from the 1000 simulated pen means per sample size.

3 Values in the range category are calculated as the true study means multiplied by the maximum difference for each sampled performance and myopathy parameter. True study means were reported as: live weight = 3.523 kg; breast yield = 25.23%; tender yield = 5.15%; wing yield = 7.57%; leg quarter yield = 22.07%; woody breast mean score = 0.74; white striping mean score = 0.95. (Maynard and Owens, 2022).

**Table 2:** Maximum percent difference in male Ross 708 performance and myopathy characteristics for different sample sizes based on a simulated 1000 pens of the true mean of all birds sampled per pen.

Sample Size^1^ (n=1000)	Live Weight Difference^2^	Live Weight Range^3^	Breast Yield Difference	Breast Yield Range	Tender Yield Difference	Tender Yield Range	Wing Yield Difference	Wing Yield Range	Leg Quarter Yield Difference	Leg Quarter Yield Range	Woody Breast Difference	Woody Breast Range	White Striping Difference	White Striping Range
5 birds	5.16	4.472-4.959	4.47	23.59-25.79	6.44	4.50-5.12	3.31	7.26-7.76	3.26	22.75-24.29	41.30	0.70-1.70	28.02	0.93-1.65
6 birds	4.49	4.504-4.928	3.90	23.73-25.65	5.52	4.54-5.08	2.86	7.29-7.73	2.82	22.86-24.18	36.20	0.77-1.63	24.50	0.97-1.60
7 birds	4.00	4.527-4.905	3.47	23.83-25.55	4.77	4.58-5.04	2.52	7.32-7.70	2.49	22.93-24.11	32.13	0.81-1.59	21.54	1.01-1.56
8 birds	3.49	4.551-4.881	3.09	23.93-25.45	4.21	43.61-5.01	2.26	7.34-7.68	2.22	23.00-24.04	27.84	0.87-1.53	19.13	1.04-1.53
9 birds	3.15	4.568-4.864	2.76	24.01-25.37	3.75	4.63-4.99	2.02	7.36-7.66	1.97	23.06-23.98	24.57	0.91-1.49	15.91	1.08-1.49
10 birds	2.84	4.582-4.850	2.47	24.08-25.30	3.38	4.65-4.97	1.80	7.37-7.65	1.78	23.10-23.94	22.48	0.93-1.47	15.04	1.09-1.48
11 birds	2.52	4.597-4.835	2.21	24.15-25.23	3.07	4.66-4.96	1.62	7.39-7.63	1.61	23.14-23.90	20.43	0.95-1.45	13.34	1.11-1.46
12 birds	2.26	4.610-4.822	1.96	24.21-25.17	2.73	4.68-4.94	1.43	7.40-7.62	1.42	23.19-23.85	17.85	0.99-1.41	12.29	1.13-1.44
13 birds	1.99	4.622-4.810	1.71	24.27-25.11	2.50	4.69-4.93	1.26	7.42-7.60	1.25	23.23-23.81	15.82	1.01-1.39	10.70	1.15-1.42
14 birds	1.75	4.633-4.798	1.49	24.32-25.06	2.17	4.71-4.91	1.09	7.43-7.59	1.08	23.27-23.77	13.85	1.03-1.37	8.83	1.17-1.40
15 birds	1.49	4.646-4.786	1.26	24.38-25.00	1.89	4.72-4.90	0.93	7.44-7.58	0.93	23.30-23.74	11.76	1.06-1.34	7.61	1.19-1.38
16 birds	1.23	4.658-4.774	1.03	24.44-24.94	1.58	4.73-4.89	0.75	7.45-7.57	0.75	23.34-23.70	9.57	1.09-1.31	6.23	1.21-1.37

1 Sampling of pens ranged from 5 to 20 broilers per pen processed to provide the variation possible between the subset being sampled and the true population mean.

2 Values in the difference category are presented as a percentage (%). Values are based on the maximum difference between the true observed mean of all birds processed per pen and the 5% or 95% percentiles from the 1000 simulated pen means per sample size.

3 Values in the range category are calculated as the true study means multiplied by the maximum difference for each sampled performance and myopathy parameter. True study means were reported as: live weight = 4.716 kg; breast yield = 24.69%; tender yield = 4.81%; wing yield = 7.51%; leg quarter yield = 23.52%; woody breast mean score = 1.20; white striping mean score = 1.29. (Maynard et al., 2023d).

Due to the confines of this analysis, and the data available from this experiment, simulation of pens to provide 1000 unique combinations of birds was limited to a sample size of 20 for females and a sample size of 16 for males. Therefore, this analysis will consider processing samples for both females and males in a range of ∼20% and ∼28% (5 birds) to ∼75 and ∼89% (20 and 16 birds, respectively) of a pen based on the original data.

## Results and discussion

**Fig. 1:**
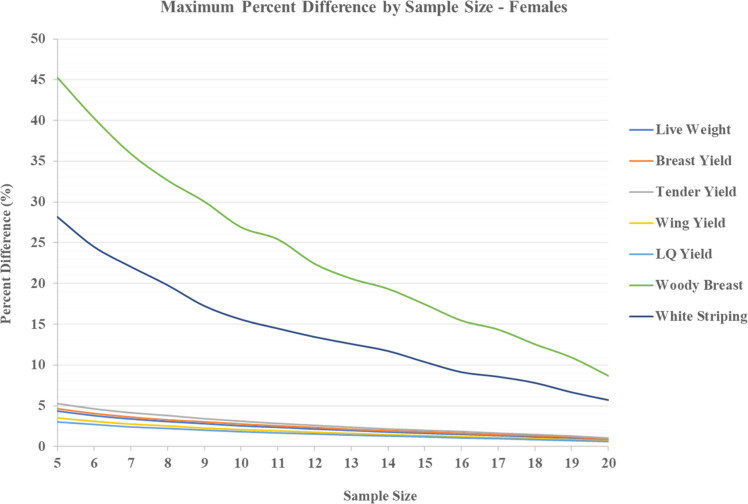
Maximum percent difference (%) of performance and myopathy characteristics for 1000 simulated pens of Ross 708 females compared to the true mean of all birds processed per pen as sample size increases from 5 to 20. Values are expressed from 5 birds sampled per pen (∼20% of pen) to 20 birds sampled per pen (∼75% of pen).

**Fig. 2:**
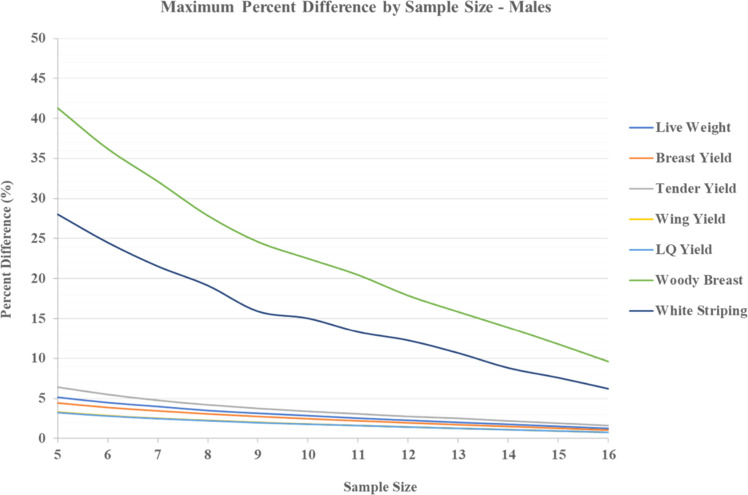
Maximum percent difference (%) of performance and myopathy characteristics for 1000 simulated pens of Ross 708 males compared to the true mean of all birds processed per pen as sample size increases from 5 to 16. Values are expressed from 5 birds sampled per pen (∼27% of pen) to 16 birds sampled per pen (∼89% of pen).

The aim of the current analysis was to better comprehend the variation presented in the literature amongst the poultry scientific community for live performance measures and myopathy measures. More specifically, this analysis was designed to examine the minimum number of samples required in broiler trials by pen, to closely match the frequency of myopathies and performance when compared to all birds processed. As presented in Table 1 (females), there appears to be two main trends present in the current analysis. Regardless of performance characteristic (i.e., weights or yields), sampling a pen with a minimum of 5 birds, or maximizing the sample with 20 birds, provided no more than 5.30% difference between the two. Given the nature of selection, this implies that sampling for basic carcass parameters, excluding sick or runt birds, would produce minimal variation and a scientist could accurately draw conclusions on the sample. Moreover, the same trend is observed in Table 2 (males) with a maximum difference noted as 6.44% in performance characteristics. Furthermore, the difference between the simulated maxima is miniscule regarding variation that may be present amongst studies that are regularly published allowing for cross examination within the literature, regardless of sex. Within common growth specific experiments, standard error of the mean can range from 1-6% with as few as four birds per pen sampled (Maynard et al., 2022c; 2023c; Ruff et al., 2021). Values in this range are highly possible based on the current findings, as the observed maximum difference when five birds processed per pen are within ∼1-2% of variation than those from the above studies, regardless of sex derived data. However, myopathy differences do not appear to follow a trend of this nature. Comparing both myopathy metrics (WB and WS) in the current analysis, there is a much larger separation of possibilities in either category. Specific to the WB aspect, variation is as high as ∼45% when a low sample of birds (i.e., 5) are processed and scored for females and as high as ∼41% when a low sample of birds are processed and scored for males (Table 1 & 2). For WS, similarities are present between females and males with a maximum difference of ∼28%. In addition, the maximum difference appears to reduce as the number of sampled birds increases. Reducing variation could then greatly influence final conclusions from a scientific experiment. If a scientist was aiming to reduce the difference of sampled birds to that of all birds at a level of 10%, sampling roughly 75% of the pen (20 birds of a 25 bird pen) would be necessary. A key piece of information regarding muscle myopathies should be pondered in this instance. Che et al. (2022) mentioned that breast myopathies (WB and WS) can occur in prevalence from 36-96% when assessing a commercial line of broilers. Wide range occurrences of this nature make the establishment of these myopathies hard to distinguish when only a small sample is processed. Sporadic distributions are hard to consider when a small sample of data are analyzed in performance-based studies rather than larger samplings seen in myopathy-based experiments. Investigation into the root cause of these myopathies are warranted, yet data suggest that small sampling practices may not be best for characterizing myopathy occurrence in research. In addition, Fig. 1, Fig. 2 depict the trend in maximum difference as sample size increases for females and males, respectively. The overall trend suggests that even as sample size increases, a tenfold difference in performance parameters and myopathy parameters is present. Again, if reducing the maximum difference to less than 10% is the specified target, sample size will need to drastically increase to meet this goal. Furthering this notion, Table 3 provides a summation of recent studies investigating performance parameters and included meat quality data collapsed as pen occurrence rates. Observing the average response of error present amongst samples, variation can range as high as 120% to as low as 11% of the pen expressing woody breast, regardless of sex. Table 3 also includes the number of birds processed per pen and is arranged by increasing percentage of pen processed. For male broilers, a numeric trend can be observed of decreasing percent error as the portion of the pen that is processed increases. This trend does not appear to be present in the limited mixed sex and female data. Therefore, the limited examples present in the literature appear to support the findings of the current analysis.

**Table 3:** Pen slaughter percentage and standard error of woody breast incidence reported in nutrition trials.

							Woody breast distribution^1^									
Trials	Broiler strain	Sex	Processing age (d)	Replicates	Birds/pen	Birds processed/pen	Percent Processed per pen (%)	0	SEM	1	SEM	2	SEM	3	SEM	Average
Ennis et al., 2020	Ross 708	Male	45	10	23	4	17.4	51.67	14.98	30.83	23.93	14.58	37.15	2.92	90.99	41.76
Ennis et al., 2020	Ross 708	Male	56	10	23	4	17.4	57.50	13.08	26.25	25.87	11.67	43.18	4.17	74.56	39.17
Maynard et al., 2022a	Cobb MV × 500	Male	33	8	21	4	19.0	83.86	6.48	11.98	40.45	4.19	81.78			42.90
Maynard et al., 2022b	Cobb MV × 500	Male	46	8	21	4	19.0	57.03	15.16	26.83	28.62	16.15	40.28			28.02
Brown et al., 2020	Ross 708	Male	60	10	20	4	20.0	4.17	76.02	25.83	26.21	40.42	18.95	25.83	25.36	36.64
Bodle et al., 2018	High-yielding	Male	46	11	30	7	23.3	8.05	18.54	42.76	5.92	36.10	7.18	13.28	14.41	11.51
Maynard et al., 2021	Cobb MV × 500	Male	36	6	21	6	28.6	62.99	12.05	25.90	28.71	11.11	46.78			29.18
Maynard et al., 2020b	Cobb MV × 500	Male	36	5	20	6	30.0	69.10	11.04	22.96	27.74	8.06	63.40			34.06
Maynard et al., 2019	Cobb MV × 700	Male	47	12	12	4	33.3	30.20	17.08	40.86	11.59	28.93	18.96			15.88
Maynard et al., 2023a	Mixed	Male	38	6	25	12	48.0	65.29	10.14	32.94	20.16	1.77	88.70			39.67
Maynard et al., 2023c	Ross 708	Male	50	12	22	13	59.1	41.03	10.83	37.50	9.95	21.48	14.44			11.74
Maynard et al., 2023a	Mixed	Male	47	6	13	12	92.3	6.74	44.21	73.10	8.07	20.17	26.67			24.32
Maynard et al., 2023d	Ross 708	Male	56	10	18	18	100.0	33.62	9.86	40.88	8.97	25.51	14.49			11.11
Maynard et al., 2022a	Cobb MV × 500	Female	33	8	21	4	19.0	97.40	2.84	2.09	122.11	0.52	245.38			123.44
Maynard et al., 2020b	Cobb MV × 500	Female	35	6	21	6	28.6	93.33	3.69	6.67	51.57					27.63
Maynard et al., 2021	Cobb MV × 500	Female	36	6	21	6	28.6	86.74	6.67	9.45	58.52	3.82	95.81			53.67
Maynard et al., 2020b	Cobb MV × 500	Female	36	5	20	6	30.0	88.30	6.85	10.27	58.93	1.46	1.37			22.38
Maynard et al., 2023a	Mixed	Female	40	6	25	12	48.0	29.79	20.21	70.04	8.60	0.17	288.24			105.68
Maynard et al., 2023a	Mixed	Female	54	6	13	12	92.3	8.74	37.30	60.77	11.14	30.49	23.02			23.82
Lemons et al., 2021	Mixed	Mixed	63	9	20	4	20.0	34.50	6.38	30.17	8.62	21.17	8.50	12.33	17.84	10.34
Maynard et al., 2019	Cobb MV × 700	Mixed	47	12	12	4	33.3	33.10	14.30	38.99	13.19	27.91	18.30			15.26
Maynard et al., 2020a	Cobb MV × 700	Mixed	47	12	12	4	33.3	67.71	7.04	25.70	17.96	6.60	38.08			21.03
Jia et al., 2022	Ross × Ross 708	Mixed	44	8	14	5	35.7	62.44	6.51	18.42	22.05	11.00	30.58	8.14	41.34	25.12
Maynard et al., 2022b	Cobb MV × 500	Mixed	53	8	14	6	42.9	51.74	12.75	32.64	17.06	15.63	32.19			20.67
Hiltz et al., 2023	Mixed	Mixed	43	3	25	12	48.0	56.67	18.60	27.50	39.28	15.83	37.22			31.70
Hiltz et al., 2023	Mixed	Mixed	57	3	13	12	92.3	34.17	31.43	38.34	21.52	26.67	24.61			25.85

1 SEM value represents SEM divided by trial average value times 100 to give a percentage value from mean

The key to consistency in production is providing a baseline for selection of criteria prior to processing (Berndtson, 1991). The current analysis further allows for predictability in selecting samples for synergism between production and quality. Another key point included in this study is that composition of results are selected on pen estimates. As mentioned previously, data is typically analyzed on a bird basis for mean score analysis in meat quality papers (Aldridge et al., 2022; Córdova-Noboa et al., 2018; Kuttappan et al., 2013; Maynard et al., 2023a). Given the nature of the experimental unit, adjusting the measure from the pen to the individual bird will greatly influence the results through error calculation and inherently “stacking” sample size to reduce experimental error. Setting the experimental unit to be consistent on performance and quality may then lead to better analyzed results as unbiased error rate will be reduced for all parameters rather than for each input considered. In addition, growing studies typically analyze the final effect on where the treatment is last applied, which is typically the pen. Therefore, proper statistical analysis would suggest that data be analyzed in this nature rather than pseudo replicating within individual pens. However, the analyzed average by pen is important for discussion. The more samples processed per pen lead to a stronger average and reduce the overall variability in the sample. Therefore, the number of birds constituting a pen average is important when cross comparing study results or factoring the bird as the experimental unit.

Implications of this study were present. The current analysis selected broilers that were used to identify stocking density requirements and dietary alterations on processing which may have altered the final WB and/or WS findings. The literature suggests that meat quality defects such as WB increase as body weight increases (Chatterjee et al., 2016; Kuttappan et al., 2012a; Maynard et al., 2023a; Tijare et al., 2016). Therefore, the inference that reduced stocking density, or dietary supplementation, produces a higher body weight may inherently increase the myopathy occurrence of the current data. For transparency, breast myopathy defects may also present themselves as a random chance per individual broiler selected. Thus, randomization of samples selected for processing may be beneficial at time of placement to more accurately determine occurrence patterns in sampled broilers, if the population cannot be collected to avoid bias when sampling at the end of the trial. An identification of individuals for sampling could lead to better understanding the sporadic nature of muscle myopathies in broilers. Further work in this area of data analysis and randomization at placement could potentially provide a better understanding of quality and performance. Furthermore, the use of a statistician in agricultural based experiments may provide a better understanding of the needs on data analysis prior to conducting research. The use of a priori may provide that there is a need for a specified level of power needed for an analysis to be conducted to provide both performance and myopathy parameters (Aaron and Hays, 2004).

For the current analysis, preliminary findings present some important characteristics to the scientific community. Being able to better understand the relationship between sampling needs and available resources will greatly impact the selection criteria for processing broilers. If performance is the only measurable data point of interest, minimum sampling requirements can be used while increasing replications to possibly increase statistical significance of treatment. Contrarily, if myopathies are the factored assessment, sampling broilers per pen will need to increase to improve the possibility of matching the true observed response with as high as 75% of the pen being processed to reduce critical error. Sampling fewer birds per pen (4-6) can lead to inaccurate assessment of myopathies.

## CRediT authorship contribution statement

**C.J. Maynard:** Data curation, Formal analysis, Writing – original draft. **K. Thompson:** Formal analysis, Supervision, Validation. **A. Mauromoustakos:** Formal analysis, Writing – review & editing. **C.M. Owens:** Conceptualization, Data curation, Formal analysis, Methodology, Project administration, Supervision, Writing – review & editing.

## Disclosures

There is no conflict of interest.

## References

[bib0001] Aaron D.K., Hays V.W. (2004). How many pigs? Statistical power considerations in swine nutrition experiments. J. Anim. Sci..

[bib0002] Aldridge D.J., Owens C.M., Maynard C., Kidd M.T., Scanes C.G. (2022). Impact of light intensity or choice of intensity on broiler performance and behavior. J. Appl. Poult. Res..

[bib0003] Berndtson W.E. (1991). A simple, rapid and reliable method for selecting or assessing the number of replicates for animal experiments. J. Anim. Sci..

[bib0004] Bodle B.C., Alvarado C., Shirley R.B., Mercier Y., Lee J.T. (2018). Evaluation of different dietary alterations in their ability to mitigate the incidence and severity of woody breast and white striping in commercial male broilers. Poult. Sci..

[bib0005] Brown A.T., Cantley S., Gutierrez O., Lemons M.E., Wamsley K.G.S. (2020). Effects of varying diet nutrient density and enzyme inclusion strategy for Ross 708 male broilers under a natural disease challenge. J. Appl. Poult. Res..

[bib0006] Chatterjee D., Zhuang H., Bowker B.C., Rincon A.M., Sanchez-Brambila G. (2016). Instrumental texture characteristics of broiler pectoralis major with the wooden breast condition. Poult. Sci..

[bib0007] Che S., Wang C., Varga C., Barbut S., Susta L. (2022). Prevalence of breast muscle myopathies (spaghetti meat, woody breast, white striping) and associated risk factors in broiler chickens from Ontario Canada. PLoS. One.

[bib0008] Coon C.N., Becker W.A., Spencer J.V. (1981). The effect of feeding high energy diets containing supplemental fat on broiler weight gain, feed efficiency, and carcass composition. Poult. Sci..

[bib0009] Córdova-Noboa H.A., Oviedo-Rondón E.O., Sarsour A.H., Barnes J., Ferzola P., Rademacher-Heilshorn M., Braun U. (2018). Performance, meat quality, and pectoral myopathies of broilers fed either corn or sorghum based diets supplemented with guanidinoacetic acid. Poult. Sci..

[bib0010] Ennis C.E., Jackson M., Gutierrez O., Cantley S., Wamsley K.G.S. (2020). Phytase and carbohydrase inclusion strategies to explore synergy within low-energy diets to optimize 56-day male broiler performance and processing. J. Appl. Poult. Res..

[bib0011] Hiltz J.Z., Maynard C.W., Anthony N.B., Orlowski S.K. (2023). Preparation.

[bib0012] Jia L., Zhang X., Li X., Schilling W., Peebles E.D., Kiess A.S., Zhai W., Zhang L. (2022). Bacitracin, bacillus subtilis, and Eimeria spp. challenge exacerbates woody breast incidence and severity in broilers. Poult. Sci..

[bib0013] Kidd M.T., McDaniel C.D., Branton S.L., Miller E.R., Boren B.B., Fancher B.I. (2004). Increasing amino acid density improves live performance and carcass yields of commercial broilers. J. Appl. Poult. Res..

[bib0014] Kuttappan V.A., Goodgame S.D., Bradley C.D., Mauromoustakos A., Hargis B.M., Waldroup P.W., Owens C.M. (2012). Effect of different levels of dietary vitamin E (DL-α-tocopherol acetate) on the occurrence of various degrees of white striping on broiler breast fillets. Poult. Sci..

[bib0015] Kuttappan V.A., Huff G.R., Huff W.E., Hargis B.M., Apple J.K., Coon C.N., Owens C.M. (2013). Comparison of hematologic and serologic profiles of broiler birds with normal and severe degrees of white striping in breast fillets. Poult. Sci..

[bib0016] Kuttappan V.A., Lee Y.S., Erf G.F., Meullenet J.F., Mckee S.R., Owens C.M. (2012). Consumer acceptance of visual appearance of broiler breast meat with varying degrees of white striping. Poult. Sci..

[bib0017] Lemons M.E., Brown A.T., McDaniel C.D., Moritz J.S., Wamsley K.G.S. (2021). Starter and carryover effects of feeding varied feed form (FF) and feed quality (FQ) from 0-18 d on performance and processing for two broiler strains. J. Appl. Poult, Res..

[bib0018] Maynard C.J., Jackson A.R., Caldas-Cueva J.P., Mauromoustakos A., Kidd M.T., Rochell S.J., Owens C.M. (2023). Meat quality attributes of male and female broilers from four commercial strains processed for two market programs. Poult. Sci..

[bib0020] Maynard C.J., Maynard C.W., Nelson D., Rochell S.J., Owens C.M. (2023). Sparing effects of dietary energy and reducing broiler breast myopathies when utilizing guanidinoacetic acid in Ross 708 males. Poult. Sci..

[bib0021] Maynard C.J., Nelson D.S., Rochell S.J., Owens C.M. (2023). Reducing broiler breast myopathies through supplementation of guanidinoacetic acid in broiler diets. J. Appl. Poult. Res..

[bib0022] Maynard C.J., Owens C.M. (2022). Effects of broiler stocking density on growth perfromance and muscle myopathies in ross 708 female broilers. Poult Sci. E.

[bib0023] Maynard C.W., Latham R.E., Brister R., Owens C.M., Rochell S.J. (2019). Effects of dietary energy and amino acid density during finisher and withdrawal phases on live performance and carcass characteristics of Cobb MV × 700 broilers. J. Appl. Poult. Res..

[bib0024] Maynard C.W., Latham R.E., Brister R., Owens C.M., Rochell S.J. (2020). Effects of dietary amino acid regimens on live performance and processing characteristics of Cobb MV × 700 male and female broilers. J. Appl. Poult. Res..

[bib0025] Maynard C.W., Liu S.Y., Lee J.T., Caldas J., Diehl E.J.J., Rochell S.J., Kidd M.T. (2020). Determining the 4th limiting amino acid in low crude protein diets for male and female Cobb MV × 500 broilers. Br. Poult. Sci..

[bib0026] Maynard C.W., Liu S.Y., Lee J.T., Caldas J.V., Diehl J.J.E., Rochell S.J., Dridi S., Kidd M.T. (2021). Determination of digestible valine requirements in male and female Cobb 500 broilers. Anim. Feed. Sci. Technol..

[bib0027] Maynard C.W., Mullenix G.J., Maynard C.J., Lee J.T., Rao S.K., Butler L.D., Hiltz J.Z., Orlowski S.K., Kidd M.T. (2022). Failure of excess leucine to impact live performance and carcass traits in male and female Cobb MV × 500 broilers during a 15- to 32-day grower period. J. Appl. Poult. Res..

[bib0028] Maynard C.W., Mullenix G.J., Maynard C.J., Lee J.T., Rao S.K., Butler L.D., Orlowski S.K., Kidd M.T. (2022). Interactions of the branched-chain amino acids. 2. Practical adjustments in valine and isoleucine. J. Appl. Poult. Res..

[bib0029] Maynard C.W., Mullenix G.J., Maynard C.J., Wells-Crafton S.C., Lee J.T., Rao S.K., Butler L.D., Orlowski S.K., Kidd M.T. (2022). Titration of dietary isoleucine and evaluation of branched-chain amino acid levels in female Cobb 500 broilers during a 22- to 42-day finisher period. J. Appl. Poult. Res..

[bib0030] Mullenix G.J., Maynard C.J., Owens C.M., Rochell S.J., Bottje W.G., Brister R.D., Kidd M.T. (2022). Spirulina platensis meal inclusion effects on broilers fed a reduced protein diet. J. Appl. Poult. Res..

[bib0031] Ruff J., Tellez G., Forga A.J., Señas-Cuesta R., Vuong C.N., Greene E.S., Hernandez-Velasco X., Uribe A.J., Martínez B.C., Angel-Isaza J.A., Dridi S., Maynard C.J., Owens C.M., Hargis B.M., Tellez-Isaias G. (2021). Evaluation of Three Formulations of Essential Oils in Broiler Chickens under. Cyclic Heat Stress. Animals.

[bib0032] Skinner J.T., Waldroup A.L., Waldroup P.W. (1992). Effects of dietary amino acid level and duration of finisher period on performance and carcass content of broilers forty-nine days of age. Poult. Sci..

[bib0033] Tijare V.V., Yang F.L., Kuttappan V.A., Alvarado C.Z., Coon C.N., Owens C.M. (2016). Meat quality of broiler breast fillets with white striping and woody breast muscle myopathies. Poult. Sci..

